# Quantum Chemical Topology Analysis of Covalent Interactions in the Hydration of F^–^ along with the Zinc Finger of NPL4 and Its Application to the Delimitation of QM/MM Boundaries

**DOI:** 10.1021/acsomega.5c11425

**Published:** 2026-03-10

**Authors:** Cristian E. Bahena-Méndez, Humberto Saint-Martin, Clare McCabe, José Manuel Guevara-Vela, Tomás Rocha-Rinza

**Affiliations:** † Instituto de Química, Universidad Nacional Autónoma de México, Circuito Exterior, Ciudad Universitaria, Delegación Coyoacán, CP 04510 Ciudad de México, México; ‡ Instituto de Ciencias Físicas, Universidad Nacional Autónoma de México, Cuernavaca, Morelos 62210, México; § School of Engineering and Physical Sciences, 3120 Heriot-Watt University, Edinburgh EH14 4AS, Scotland, U.K.; ∥ Department of Chemical and Biomolecular Engineering, Vanderbilt University, Nashville, Tennessee 37235-1826, United States

## Abstract

The accurate modeling of metalloproteins remains a central challenge in computational chemistry, particularly when classical force fields fail to describe metal–ligand interactions with significant covalent character. Herein, we present a diagnostic methodology to delimit quantum mechanical regions in hybrid quantum mechanics/molecular mechanics (QM/MM) simulations, considering (i) the hydration of F^–^ and (ii) the zinc finger (ZF) domain of the Nuclear Protein Localization 4 (NPL4) as case studies. Our approach is grounded in methods of wave function analysis within quantum chemical topology. Specifically, we rely on the quantum theory of atoms in molecules and the interacting quantum atoms approaches to partition interaction energies between (i) the F^–^ anions and solvating water molecules and (ii) the Zn^2+^ and its surrounding amino acids within the ZF of NPL4 into classical (ionic) and exchange–correlation (covalent) components. The presented methodology provides also a rational criterion to determine either the inclusion or exclusion of water molecules within the QM region as Zn^2+^···OH_2_ interactions might be relevant as indicated by electronic structure geometry optimizations. Our diagnostic offers a systematic way to define suitable QM/MM boundaries, and it is useful to decide on the inclusion of solvent or other species in the QM region of hybrid simulations when covalent contributions are uncertain or only partially present. This analysis reveals that (i) the first solvation layer of F^–^ and (ii) the first coordination shell of Zn^2+^ exhibit significant covalency in their interaction with the F^–^ and Zn^2+^ ions, respectively, justifying a minimal QM region comprising three cysteines, one histidine, and the metal center for the ZF in NPL4. The QM/MM hybrid simulations for the hydration of the F^–^ anion showed important differences with respect to classical molecular mechanics concerning the coordination number and maxima of F^–^···O peaks in the radial distribution functions. Hybrid QM/MM simulations using this covalency-based region in the ZF of NPL4 recover accurate coordination geometries and bond lengths, correcting important distortions introduced by classical force fields, including spurious hydration and hypercoordination of the Zn^2+^ center. Both the enlargement and reduction of the QM region result in incorrect coordination geometries and less accurate Zn^2+^–ligand distance distributions. The diagnostic method put forward herein shows that metrics based on the chemical bonding scenario can inform the construction of transferable QM regions for the solvation of ions and metalloproteins. Overall, our results establish a general protocol to integrate wave function analysis with hybrid modeling, and they highlight the potential of such analysis to improve the accuracy of QM/MM simulations.

## Graphical abstract

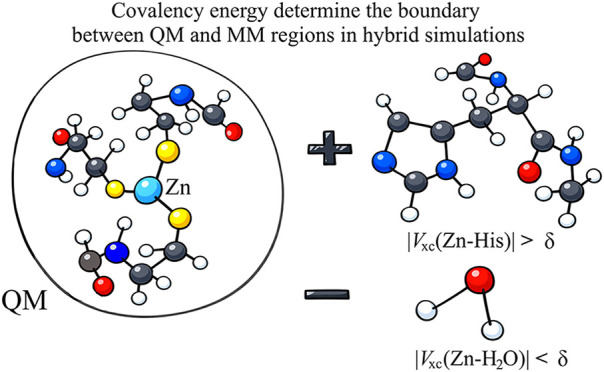

## 1 Introduction

Molecular dynamics (MD) simulations have been widely employed to investigate metalloproteins,^1–7^ yet classical force fields often fail to adequately describe metal–ligand coordination, especially for transition metals.^1–3,8,9^ These limitations occur mostly because of fixed charge models and spherically symmetric interaction potentials that do not account for the directionality or the covalent character of coordinate bonds between the investigated metal centers and their ligands.^10,11^ Hybrid quantum mechanics/molecular mechanics (QM/MM) calculations often provide a more accurate alternative for the study of metal dative bonds than classical MD simulations. For example, QM/MM computations improve the description of coordination environments for transition ion metals such as Cd^2+^, Zn^2+^, Cu^2+^, and Hg^2+^ in comparison with classical MD.^12–15^ Another case regarding the better accuracy of QM/MM computations with respect to classical molecular simulations is the fact that although classical MD simulations with three-body corrections can provide an adequate evaluation of structural data of hydrated transition metals, they are not accurate enough to describe certain properties of these systems.^12–15^ For example, the proper description of hydration energies and ion–ligand vibrations requires quantum mechanical corrections.^13,14^ The account of effects like the Jahn–Teller distortion in the hydration of Cu^2+^ demands a quantum mechanical treatment as well.^12^ Moreover, unlike MM simulations, QM/MM calculations are highly effective for modeling and thermodynamic studies. For instance, the hydration of the Zn^2+^ cation described by QM/MM simulations shows a good agreement with experimentally determined structural and thermodynamic data.^14^ Furthermore, QM/MM calculations predict relative solvation free energies more accurately than classical models when compared to experimentalresults.^14^ As a final example, QM/MM reproduces the experimental result that Zn^2+^ has a significantly more negative solvation free energy than Mg^2+^, despite both cations having the same net charge and generally similar solvent structures.^14^

The inclusion of only the first coordination shell is often sufficient for an accurate description of the above-mentioned effects using QM/MM simulations, although the incorporation of the second shell can further improve such description.^12–14^ Indeed, the reliability and accuracy of hybrid QM/MM simulations depend critically on how the QM region is defined.^16–19^ For the purpose of delimiting QM and MM regions in hybrid simulations, some approaches take advantage of the convergence behavior of certain properties to define the optimal QM/MM boundary, aiming to balance accuracy with computational efficiency. While increasing the size of the QM region can lead to more accurate results in some cases, the concomitant computational cost increases significantly. Therefore, it is desirable to identify a QM region of reasonable size that still captures the essential physics and chemistry of the investigated system. It is well known that energetic and structural properties tend to approach asymptotic limits only when very large QM regions are used (ca. 500–1000 atoms).^20–24^ One of the main factors underlying this slow convergence is the charge transfer between the QM region and the surrounding protein environment.^25^ Therefore, an efficient strategy is needed to determine an optimal QM region while gaining insights into the crucial interactions that are captured only with large QM models. One approach for this purpose is that proposed by Karelina and Kulik,^26^ who coupled two complementary techniques, i.e., charge shift analysis (CSA) and Fukui shift analysis (FSA). The CSA methodology takes into account changes in atomic partial charges which are summed over residues (RES) under two different circumstances, i.e., the consideration of (i) the complete enzyme (holo) and (ii) a protein deprived of its active site (apo), so that the charge shift may be calculated as,

1 $$\Delta q_{\mathrm{RES}}=q_{\mathrm{RES}}^{\mathrm{holo}}-q_{\mathrm{RES}}^{\mathrm{apo}}$$

Similarly, FSA is based on the summation of condensed Fukui functions over a fragment, i.e.,

2 $$f_{+}^{\mathrm{RES}}=\sum_{i\in \mathrm{RES}}q_{i}\left(N+1\right)-q_{i}\left(N\right)$$

3 $$f_{-}^{\mathrm{RES}}=\sum_{i\in \mathrm{RES}}q_{i}\left(N\right)-q_{i}\left(N-1\right)$$

under two different contexts, namely, with and without the addition of an extra residue. Despite their promising features, CSA and FSA present a few inconveniences. We point out first that the underlying physics of CSA and FSA methods, i.e., changes in atomic charges and condensed Fukui functions to determine the QM region in hybrid simulations, are ultimately reflected in the polarization of the electron density. Such polarization might be accounted for to some extent by classical polarizable force fields. Nevertheless, these approaches do not consider the magnitude of the covalent interactions. We stress the relevance of this point because covalency is a quantum mechanical effect that cannot be taken into consideration to any extent by classical force fields. The proper description of covalent interactions is important even for systems wherein the formation or destruction of covalent bonds does not occur because the adequate description of charge density within regions governed by covalent interactions can influence and correct the description of the MM region. For instance, the correct description of the first hydration shell in the hydration of the Cu^2+^ ion (QM region) leads to an improved description of the second shell (MM region), which is not explicitly included in the QM treatment.^12^

Another inconvenience of the CSA and FSA approaches is their reliance on charge population analysis methods such as that put forward by Mulliken, which might depend strongly on technical aspects of the calculation (e.g., the choice of basis set).^27^ Furthermore, for the sake of efficiency and numerical robustness, the implementation of CSA and FSA was based on Voronoi Deformation Density (VDD) partial charges.^28^ While VDD charges are reasonably insensitive to the choice of basis sets,^28^ the Voronoi cells are merely geometric constructs not supported by any theory or method.^29^ Regarding FSA, the condensed Fukui function has other limitations^30^ which are particularly relevant for the determination of the QM region in hybrid QM/MM simulations. For example, the FSA approach requires the use of density functional approximations that have asymptotically correct exchange. Moreover, delocalization errors can lead to inaccuracies of the Kohn–Sham molecular orbitals, which might affect the computation of the condensed Fukui function, leading to false positives.^26^ Another shortcoming of the condensed Fukui function, which is considerably important for this investigation, is its unsuitability for the treatment of transition metal complexes.^31^

The main difference between the QM and MM regions in hybrid QM/MM simulations is the treatment of chemical interactions. Therefore, it seems sensible to exploit state-of-the-art techniques for the examination of the chemical bonding scenarios to determine these regions. More specifically, because covalency is essentially a quantum mechanical effect as mentioned above, we consider the covalent contribution to the interactions of interest within QM areas of hybrid QM/MM simulations as the basis for the division of QM and MM regions. The accounting of covalency is a main distinctive feature of this investigation with respect to other methodologies to determine QM subsystems in QM/MM hybrid simulations, e.g., CSA and FSA. In this work, we developed and applied a methodology based on wave function analysis in the field of Quantum Chemical Topology (QCT) to define the QM region in hybrid simulations of (i) the aqueous solvation of the F^–^ anion and (ii) the Zinc Finger (ZF) of the Nuclear Protein Localization 4 (NPL4) which we took as case studies. We selected these systems as suitable examples to illustrate the methodology put forward herein. Indeed, the determination of the QM region in hybrid QM/MM simulations for these case studies involves choosing (i) the inclusion of solvation water molecules for the F^–^ hydration and the Zn finger given the external character of this system and (ii) the amino acids surrounding the Zn^2+^ center. We exploited the Quantum Theory of Atoms in Molecules (QTAIM) to delimit the boundaries of atomic systems within the system under investigation, as the QTAIM partition is without a doubt the one that has the greatest number of virtues from a theoretical point of view,^29^ e.g., concerning the computation of the expectation values of atomic kinetic energies.^32^ We also used the Interacting Quantum Atoms (IQA) partition of the electronic energy to quantify the covalent character of F^–^···H–OH and Zn^2+^···ligand interactions.[Fn fna] With this information at hand, we delimited the QM subsystem and performed QM/MM simulations employing the GFN2-xTB^33^ method for the QM region and standard force fields for the remainder of the system for both the analysis of the hydration of F^–^ and the study of the Zn finger in NPL4. Specifically, we implemented an adaptive QM/MM scheme to dynamically account for the exchange of water molecules between the QM and MM subsystems for the F^–^ anion in aqueous solution. Regarding ZF in NPL4, we analyzed two representative scenarios for the QM region selection based on QTAIM and IQA analyses. First, (a) the QM region was defined by including only the residues whose interactions with Zn^2+^ exhibit a strong covalent component to the corresponding interaction energy. This model was further used to perform QM/MM calculations with both extensions and reductions of the QM region to assess the results of the corresponding simulations. Second, (b) the QM system was extended using the region defined in (a) along with two additional water molecules, forming an octahedral coordination around the Zn^2+^ center. The resulting coordination geometries and bond length distributions obtained from hybrid simulations using the QCT-determined QM region match more closely those from electronic structure theory and experimental data than the results obtained from classical simulations. These findings indicate that the examination of the chemical bonding scenario via the topological analysis of the electron density can serve as a rigorous, systematic, and unambiguous diagnostic tool to define QM regions in hybrid QM/MM simulations to study the hydration of ions and metalloproteins.

## 2 Theoretical Framework

We examined the electronic density, ρ­(**r**), obtained from our density functional theory (DFT) calculations concerning the hydration of F^–^ and the ZF of NPL4, using QTAIM, which employs this scalar field to partition the three-dimensional (3D) space of an electronic system into atomic basins. This approach enables the segmentation of molecules, molecular clusters, and crystalline solids into atoms and functional groups, thereby recovering classical chemical concepts such as chemical bonding and bond order.^35^ On the other hand, IQA is a valuable and versatile tool in theoretical chemistry to analyze intra- and intermolecular interactions in electronic systems without the need of a reference system.^36–41^ Both IQA and QTAIM have proven important to characterize chemical interactions of different natures on the same rigorous footing.^40,42–47^ In particular, these methods allow for a detailed assessment of the ionic and covalent contributions of interatomic interactions within electronic systems, as described below.

Regarding the general features of these methods, QTAIM has become a standard conceptual tool in molecular quantum chemistry.^36^ The QTAIM defines a theoretical framework that decomposes molecular fragments into quantum subsystems with well-defined expectation values of quantum mechanical observables. For instance, one can calculate the net atomic charge within an atomic basin Ω_A_,^48^

4 $$Q\left(\Omega_{A}\right)=Z\left(\Omega_{A}\right)-\left\langle N\left(\Omega_{A}\right)\right\rangle$$

where *Z*(Ω_A_) and ⟨*N*(Ω_A_) ⟩ are the atomic number of the nucleus and the expected number of electrons within basin Ω_A_, respectively. The value of ⟨*N*(Ω_A_)⟩ is calculated by integrating ρ­(**r**) over the volume of the corresponding basin. The computation of QTAIM charges gives valuable information about charge transfer processes.^29,49^ Moreover, QTAIM offers other valuable insights into the nature of chemical interactions. For example, the QTAIM defines the number of delocalized electrons between two atoms, denoted as delocalization index (DI),^48^

5 $$\mathrm{DI}\left(\Omega_{A},\Omega_{B}\right)=-2\mathrm{cov}\left(N\left(\Omega_{A}\right),N\left(\Omega_{B}\right)\right)$$

wherein cov­(*x*, *y*) represents the covariance between the random variables *x* and *y*. This index is identified with the number of electrons shared between two atoms; i.e., it is a measure of the contribution of covalency to the interaction between Ω_A_ and Ω_B_. QTAIM indicators are reasonably stable with respect to the electronic structure approximation or basis set employed in computing the molecular electron density of the system of interest. These indicators are also orbital invariant, as they are derived from the analysis of the electron density, which corresponds to the expectation value of a Dirac observable as opposed to CSA and FSA.^48^

The IQA analysis extends the conceptual framework established by the QTAIM spatial partitioning by introducing a decomposition of the total electronic energy of an electronic system into intra- and interatomic contributions,^34,50^

6 $$E=\sum_{A}E_{\mathrm{net}}^{A}+\sum_{A}\sum_{A<B}E_{\mathrm{int}}^{\mathrm{AB}}$$

where *E*_net_^A^ denotes the contribution of atomic basin Ω_A_ to the total electronic energy and *E*_int_^AB^ accounts for the interaction energy between atomic basins Ω_A_ and Ω_B_.

The IQA partition of the electronic energy relies on the first-order reduced density matrix, ρ_1_(**r**_1_; **r**_1_^′^), and the electron pair density, ρ_2_(**r**_1_, **r**_2_).^34^ Although DFT does not provide a method to compute any of these scalar fields, the IQA method has been implemented for Kohn–Sham electron densities.^51,52^ Equation 6 can also be expressed in terms of disjoint groups of atoms G,H,··· , which constitute the total electronic system. In other words, we can rewrite the RHS of formula [Disp-formula eq6] by considering the net energies of each of these fragments plus the interaction energies among them,^34^

7 $$E=\sum_{G}E_{\mathrm{net}}^{G}+\sum_{G}\sum_{G<H}E_{\mathrm{int}}^{\mathrm{GH}}$$

Additionally, the IQA energy partition allows us to describe the nature of intra- and intermolecular contributions by dividing the interaction energy into classical (*V*_cl_^AB^) and exchange–correlation (*V*_xc_^AB^) terms,

8 $$E_{\mathrm{int}}^{\mathrm{AB}}=V_{\mathrm{cl}}^{\mathrm{AB}}+V_{\mathrm{xc}}^{\mathrm{AB}}$$

The terms *V*_cl_^AB^ and *V*_xc_^AB^ correspond, respectively, to the ionic and covalent contributions to the interaction between atoms Ω_A_ and Ω_B_. A similar formula holds for the groups G and H . Given the fact that both *V*_xc_^AB^ and DI­(Ω_A_, Ω_B_) are associated with covalent bonding, a correlation between these two quantities can be anticipated, which is indeed the case. More specifically, the principal term in the multipole expansion of *V*_xc_^AB^ involves the index DI­(Ω_A_, Ω_B_) directly, i.e.,

9 $$V_{\mathrm{xc}}^{\mathrm{AB}}\approx -\frac{\mathrm{DI}\left(\Omega_{A},\Omega_{B}\right)}{2R},,\left(\mathrm{for}\;\mathrm{large}\;R\right)$$

wherein *R* is the distance between the nuclei within atomic basins Ω_A_ and Ω_B_. In summary, the IQA interaction energy *E*_int_^AB^ can be decomposed into ionic and covalent components, enabling a quantitative assessment of the degree of ionicity and covalency present in each pairwise interaction within electronic systems.

The most significant computational demand of the IQA analysis stems from the three- and six-dimensional integrals required to evaluate one- and two-electron properties over the typically irregular volumes defined by QTAIM atomic boundaries.^37^ Nevertheless, eq 9 leads to significant savings in computer time because it does not involve the weight factor *r*_12_^–1^ inside six-dimensional integrals, and the calculation of DI­(Ω_A_, Ω_B_) can be reduced to the computation of overlap integrals within the basins Ω_A_ and Ω_B_.^37^

## 3 Computational Details

### 3.1 Hydration Structure of the Fluoride Ion

The determination of local minima on the potential energy surfaces of X···(H_2_O)*_n_* clusters is challenging due to the large number of accessible configurations for these systems. In order to sample this landscape efficiently for F^–^···(H_2_O)*_n_* complexes, we performed global structure searches using the Gradient Embedded Genetic Algorithm (GEGA) developed by Alexandrova and co-workers,^53^ as implemented in CLUSTER.^54^ In GEGA, the genetic algorithm operators generate new candidate structures, but each candidate is then driven down the potential energy surface using ab initio gradients, so that every structure retained in the evolutionary pool corresponds to a genuine local minimum. This condition is enforced in practice via geometry optimizations followed by harmonic frequency analyses, which filter out saddle points and ensure that isomers are compared at bona fide local minima. This gradient embedded workflow reduces the number of genetic algorithm iterations typically required by conventional schemes that rely primarily on single point energies while retaining ab initio quality for the resulting geometries and relative isomer energies. The GEGA searches and local refinements were carried out with ORCA 4.2 ^55^ using the M06 2X/def2-TZVP approximation.^56,57^ The GEGA procedure used an initial population of 10 candidate structures that evolved over 8 generations. The resulting low-energy structures were then reoptimized with the B3LYP/def2-TZVP method,^57–59^ and harmonic frequency calculations were performed to confirm that the final geometries represent local minima on the corresponding potential energy surfaces.

### 3.2 Model Construction and System Preparation of the NPL4 System

Among the pertinent structures in the RCSB-PDB database, we selected that with PDB ID 6JWH^60^ (corresponding to the crystal structure of yeast NPL4) as a template of the ZF in NPL4 due to its high resolution (1.72 Å). Attention was put on the zinc-binding domain of the yeast NPL4 protein (residues 129–151), which we refer as NPL4­(Zn^2+^)_129–151_.^1^ Additional models with PDB IDs 6JWI^60^ and 6JWJ^60^ were used as templates to reconstruct missing loops, yielding a full-length and robust model of the ZF in NPL4 for classical and hybrid QM/MM simulations. The completion of structures was carried out using the program MODELER v.10.6.^61^ Protonation states were assigned using CHARMM-GUI^62–64^ and the H++ ^65^ server. Terminal capping was applied by using the −NH_2_ group at the N-terminus and a neutral carboxyl group at the C-terminus. His139 was modeled in the Nδ1–H tautomeric form. Aspartate, glutamate, arginine, and lysine residues were treated as charged, while the zinc-coordinating cysteines were considered as deprotonated. All remaining cysteine residues were modeled as protonated.

### 3.3 Quantum Mechanical Calculations and Pocket Definition for the ZF in NPL4

Geometry optimizations were performed based on the X-ray structure of NPL4­(Zn^2+^)_129–151_ (PDB ID: 6JWH^60^). Coordinating residues were truncated at the C_α_ positions, and methyl radicals were added as capping groups. Atomic coordinates were optimized using the B3LYP^58,59^ exchange–correlation functional along with the def2-TZVP^66^ basis set. Frequency analyses confirmed that the optimized structures corresponded to local minima on the relevant potential energy surfaces. The coordination environment of the metal ion was decomposed into spatial pockets based on their corresponding distance from the Zn^2+^ center. These pockets (Figure S1) are not to be confused with Regions I–III defined in Section 4.4. Pocket 1 contained the directly coordinating residues to Zn^2+^, i.e., Cys137, His139, Cys145 and Cys148. In order to consider the possible inclusion of solvating water molecules in the QM region, we extracted a representative snapshot from classical MD simulations of NPL4 using the CHARMM36m force field. In this structure, the metal center exhibits an octahedral coordination geometry composed of three cysteines, one histidine, and two water molecules. The coordinates of the ZF atoms were extracted and optimized using the semiempirical method GFN2-xTB.^33^ Regarding the accuracy of this method, ref 67 reports a benchmark study concerning the performance of tight binding approaches GFN1-xTB and GFN2-xTB in geometry optimizations with respect to the hybrid meta-GGA functional TPSSh-D3­(BJ)-ATM along with the basis set def2-TZVPP. The structures of transition metal complexes and organometallic supramolecular structures up to Hg (*Z* = 80) computed with both tight binding approaches are in good agreement with those calculated with DFT.^67^ Based on the GFN2-xTB geometry optimizations, we defined Pocket 2 as an extension of Pocket 1, including the same coordination residues with the addition of two water molecules, which coordinate to the zinc center. Pocket 3 included those of Pocket 1, and it additionally encompassed two more adjacent residues, namely, Met144 and Tyr147. Finally, Pocket 4 comprises those amino acids in Pocket 3 along with residues Ser134 and Lys138. In order to further assess the quality of the above-mentioned geometry optimizations with GFN2-xTB, we contrasted the structures of the optimized structures with this method and with B3LYP/def2-TZVP. The comparison shows that the differences between B3LYP and GFN2-xTB for Zn–S distances range from 0.03 to 0.07 Å, while for Zn–N distances, the difference is around 0.12 Å. The similarity of both structures is also revealed by the corresponding root-mean-square deviation (RMSD) of 0.20 Å and the consideration of the numerous degrees of freedom of the system. Single-point DFT calculations were performed on each pocket using the B3LYP/TZVP^58,59,68^ approximation to compute electron densities. These calculations were carried out with the program GAUSSIAN v.16,^69^ and the resulting electron densities were subsequently analyzed with the QTAIM and IQA methods of wave function analysis using the AIMALL^70^ suite of programs. We would like to point out that we used the GFN2-xTB method based on efficiency grounds and its overall good performance regarding the geometry of transition metal complexes.^67^ Unfortunately, it is not possible to carry out an IQA energy partition, not even in an approximate manner, based on this electronic structure method. Therefore, we used DFT (more specifically, B3LYP) densities to carry out the IQA analyses presented in this investigation.

### 3.4 Molecular Dynamics Simulations

Classical molecular dynamics simulations for the hydration of the F^–^ anion were performed using the 12-6-4 Lennard-Jones parameters developed by Li and Merz^71,72^ in combination with the TIP3P water model.^73^ In order to further investigate the hydration of the F^–^ anion, two adaptive QM/MM schemes were employed for the corresponding simulations. The first scheme utilized a QM region with one mobile and four fixed water molecules in the transition (active) region, thereby allowing for a maximum of five H_2_O molecules in the QM region. The second scheme followed a similar setup, but it was extended to include one mobile and six fixed H_2_O molecules, therefore exploring effectively coordination environments of up to seven H_2_O monomers. Regarding the ZF in NPL4, classical molecular dynamics simulations were performed for both the full-length NPL4 protein and the truncated NPL4­(Zn^2+^)_129–151_ domain, while hybrid QM/MM molecular dynamics calculations were carried out only for the latter system. These simulations were conducted using three force fields: CHARMM36m,^74^ FF99SB-ILDN,^75^ and FF19SB.^76^ For hybrid QM/MM simulations, the QM region was defined based on the analysis of the chemical bonding scenario of the interactions between (i) F^–^ and H_2_O molecules in the hydration of the former species and (ii) the Zn^2+^ center and its surrounding ligands, i.e., the amino acids and the solvating water molecules enclosed in Pockets 1–4 defined in Section 3.3. In particular, we focused on the covalent contributions to such interactions, as described in Section 4. The quantum mechanical subsystem was addressed using the GFN2-xTB^33^ tight binding method. Correspondingly, the MM region was studied with each of the force fields. All MD simulations[Fn fnb] were preceded by an energy minimization step, and they were performed using AMBER 24.^77^

A cubic box of 24.6 Å along with 499 explicit TIP3P water molecules under periodic boundary conditions were used to simulate the hydration of the F^–^ anion. A 5000-step optimization was performed, initiated by 2500 steps of steepest descent. A nonbonded cutoff of 10.0 Å was used for all interactions. On the other hand, the ZF in NPL4 was solvated in a cubic box of explicit TIP3P water molecules, ensuring a minimum distance of 10.0 Å between any protein atom and box edges. Periodic boundary conditions were applied in all directions. In order to neutralize the net charge of the ZF in NPL4 and to reproduce physiological ionic strength, Na^+^ and Cl^–^ ions were added to the system. The energy minimizations of ZF in NPL4 involved a maximum of 10,000 steps: the initial 1000 steps employed the steep descent method, while the remaining steps used the conjugate gradient minimization. A nonbonded cutoff of 12 Å was used for both electrostatic and van der Waals interactions, and a positional restraint of 1.0 kcal mol^–1^ Å^–2^ was applied to the protein backbone during energy minimizations.

Both the ZF in NPL4 and the hydrated F^–^ ion were then gradually heated over 100 ps with a time step of 2 fs under an NVT ensemble using Langevin dynamics,^78^ with a collision frequency γ = 2.0 ps^–1^. Throughout heating, backbone atoms were restrained with a force constraint of 10.0 kcal mol^–1^ Å^–2^. The temperature was linearly increased from 10 to 298 K. Following heating, a 20 ps equilibration was performed under NPT conditions at 298 K and 1 atm, using a Berendsen barostat^79^ (τ_p_ = 1.0 ps) and a Langevin thermostat (γ = 2.0 ps^–1^). During this phase, the force constraint on the backbone atoms was reduced to 1.0 kcal mol^–1^ Å^–2^. Finally, for the ZF in NPL4, production MD simulations without restrictions were carried out under NPT conditions using the same thermostat and barostat settings. Concerning the hydration of the F^–^ anion, simulations were performed under the NVT ensemble. The electronic structure of the QM region in adaptive QM/MM MD simulations was calculated with the GFN2-xTB approximation, while the rest of the system was described using Li and Merz parameters^71,72^ for the ion and the TIP3P model for water.^73^ To ensure numerical stability within the QM region, a reduced time step of 0.5 fs was used and no bond constraints were applied. A collision frequency of γ = 5.0 ps^–1^ was maintained via a Langevin thermostat. Classical nonbonded interactions were addressed with a 10.0 Å cutoff, while electrostatic interactions across the QM/MM interface were handled using the particle-mesh Ewald method with a specific QM cutoff of 5.0 Å. Both classical and adaptive QM/MM production runs were performed for 500 ps.

For all hybrid QM/MM MD simulations of the ZF in NPL4, the SHAKE algorithm was employed to constrain bonds crossing the QM/MM boundary. A cutoff of 10 Å was applied for nonbonded interactions, and electrostatic contacts across the QM/MM interface were considered using the particle-mesh Ewald method. Production runs were performed for 10 ns for classical MD and 5 ns for hybrid QM/MM simulations, with a 2 fs integration time step in both cases, at a constant temperature of 298 K. Analysis of coordination geometry and metal–ligand distance distributions was carried out using the software SHAPE v2.1 ^80,81^ and AMBERTOOLS v24.^77^

## 4 Results and Discussion

### 4.1 Hydration of the F^–^ Anion

First, we consider the hydration of the fluoride anion as a case study for the validation of the methodology put forward in this work. Later, we examine the ZF in NPL4 as a test system to assess our diagnostic method. In order to quantify the covalent contributions involved in the fluoride hydration, we utilized the IQA partition analysis to evaluate the covalent and ionic components in F^–^···H_2_O interactions. As reported in Table 1, the exchange–correlation contribution with the largest magnitude in the [OH_2_···F···H_2_O]^−^ cluster shows a value of −39.8 kcal mol^–1^, in contrast to the classical contribution of −28.1 kcal mol^–1^. In other words, the covalent component in the F^–^···H_2_O interaction is considerably stronger than its ionic counterpart, as previously reported by Sauza-de la Vega and co-workers.^82^ We emphasize that this result is contrary to chemical intuition, thereby showing an important field of application for the diagnostic method put forward herein. Owing to the charge of F^–^ and the large dipole moment of the H_2_O molecule, chemical intuition would suggest that the interaction should be dominated by strong classical (i.e., Coulombic) forces, e.g., charge–dipole interactions. More specifically, the covalent contribution to the interaction energy between F^–^ and water molecules amounts to 39.80 and 36.40 kcal mol^–1^ in the [OH_2_···F···H_2_O]^−^ cluster. Notably, this exchange–correlation component is stronger than the interaction between water molecules in (H_2_O)_6_ as shown in Table 1.

**Table 1:** Exchange–Correlation Energies (*E*_xc_) of Zn^2+^···OH_2_, H_2_O···H_2_O and F^–^···H_2_O Interactions in Different Environments

system	Approx.	*E*_xc_ (kcal mol^–1^)
[Zn(H_2_O)_6_]^2+^	PBE0/Def2-TZVP	–58.00
[Zn···(H_2_O)_2_]^2+^	B3LYP/TZVP	–19.35/–15.82
[OH_2_···F···H_2_O]^−^	B3LYP/Def2-TZVP	–39.80/–36.40
(H_2_O)_6_	B3LYP/TZVP	–27.53

Given the marked covalent character of the F^–^···H_2_O interaction, we carried out QM/MM calculations, wherein we included the water molecules within the first solvation shell in the QM region, and we compared the corresponding results with classical MD simulations. We characterized the structural properties of hydrated F^–^ by means of F^–^···O and F^–^···H Radial Distribution Functions (RDFs) and their corresponding coordination numbers. In this regard, classical MD simulations yield the first F^–^···O peak at 2.60 Å (Figure 1). The first hydration shell is well-separated from the second one, leading to an average coordination number of 6. In the QM/MM simulations, a broader and less pronounced first peak is observed at a larger F^–^···O distance of 2.69 Å corresponding to a first hydration shell with an average coordination number of 7 (Figure 2). The fact that the F^–^···O peak in hybrid QM/MM calculations is farther away than those corresponding to classical MD calculations is consistent with the anticooperative effects of F^–^···H_2_O interactions described previously in ref 82. The larger first hydration shell of F^–^ for hybrid QM/MM simulations in comparison with classical MD simulations is consistent with previous results of Tongraar et al.^83^ These workers reported first-peak distances of approximately 2.68 Å for QM/MM (Hartree–Fock (HF)/BJH-CF2) and 2.60 Å for classical MD. However, their reported coordination number (NC) of NC = 4.6 contrasts sharply with our result of NC = 7 as reported in Figure 2. This discrepancy arises primarily from the lack of electron correlation in the HF theory. In the absence of dispersion forces, the short-range Pauli repulsion is not balanced by attractive van der Waals interactions,^84^ resulting in an artificially hard ion description that effectively pushes solvent molecules away. In contrast, the GFN2-xTB potential provides a more polarizable and somewhat softer description of the F^–^ anion. When combined with solvent flexibility (O–H bond stretching), the system reduces steric hindrance, allowing a seventh water molecule to be stable within a dynamic first shell. This feature is typically suppressed by the rigidity of the HF approximation or classical models. The QM/MM results further indicate that water molecules in the first hydration shell are relatively mobile, facilitating an easier exchange between the first shell and the outer regions. In both classical and hybrid QM/MM simulations, a second layer of water molecules can be weakly identified (Figure 3). The classical MD simulation exhibits a first F^–^···H peak at 1.70 Å, followed by a distinct second peak at 2.99 Å, corresponding to the two hydrogen atoms of the water molecules in the first shell. The difference of approximately 0.9 Å between the first F^–^···H and F^–^···O peaks indicates a preference for linear hydrogen-bond formation. On the other hand, the first two F^–^···H peaks are shifted to shorter distances (1.61 and 2.84 Å), and they appear broader and less pronounced than the corresponding peaks in classical MD simulations. The distance between the first F^–^···H and F^–^···O peaks increases in hybrid QM/MM simulations to 1.08 Å, indicating a deviation from the linearity of the hydrogen bonds observed in classical MD calculations. This shift, together with the observed O–H bond stretching and H–O–H angle relaxation, reflects a more flexible and deformable solvation geometry. The QM/MM (GFN2-xTB) description captures a larger angular displacement and a more disordered and labile hydration complex. The nonrigid character of the GFN2-xTB potential effectively accounts for the complex interplay between the ion and its flexible aqueous environment, contrasting with the more rigid and structureless shells predicted by HF.

**Figure 1:**
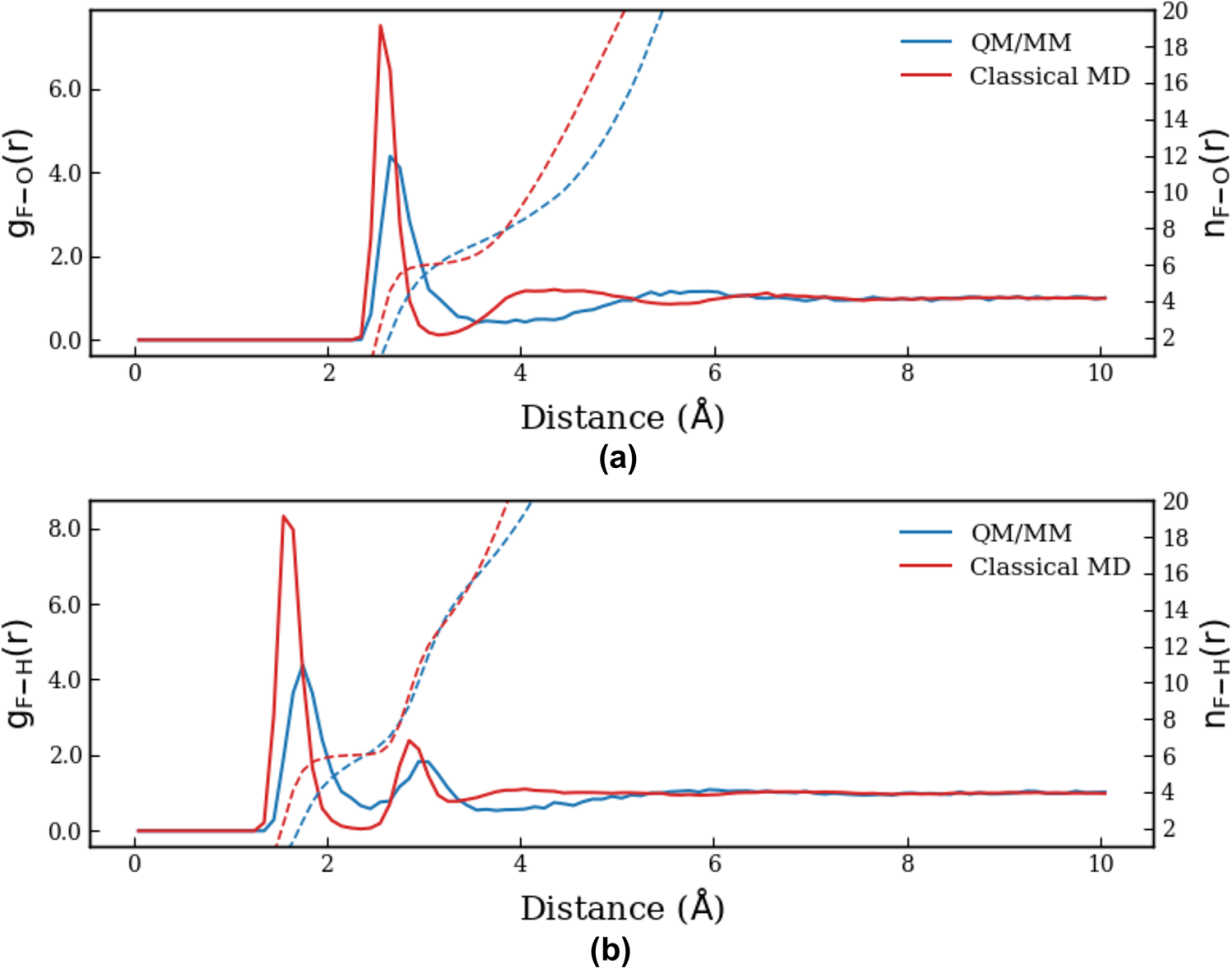
(a) F^–^···O and (b) F^–^···H radial distribution functions and their corresponding integration numbers.

**Figure 2:**
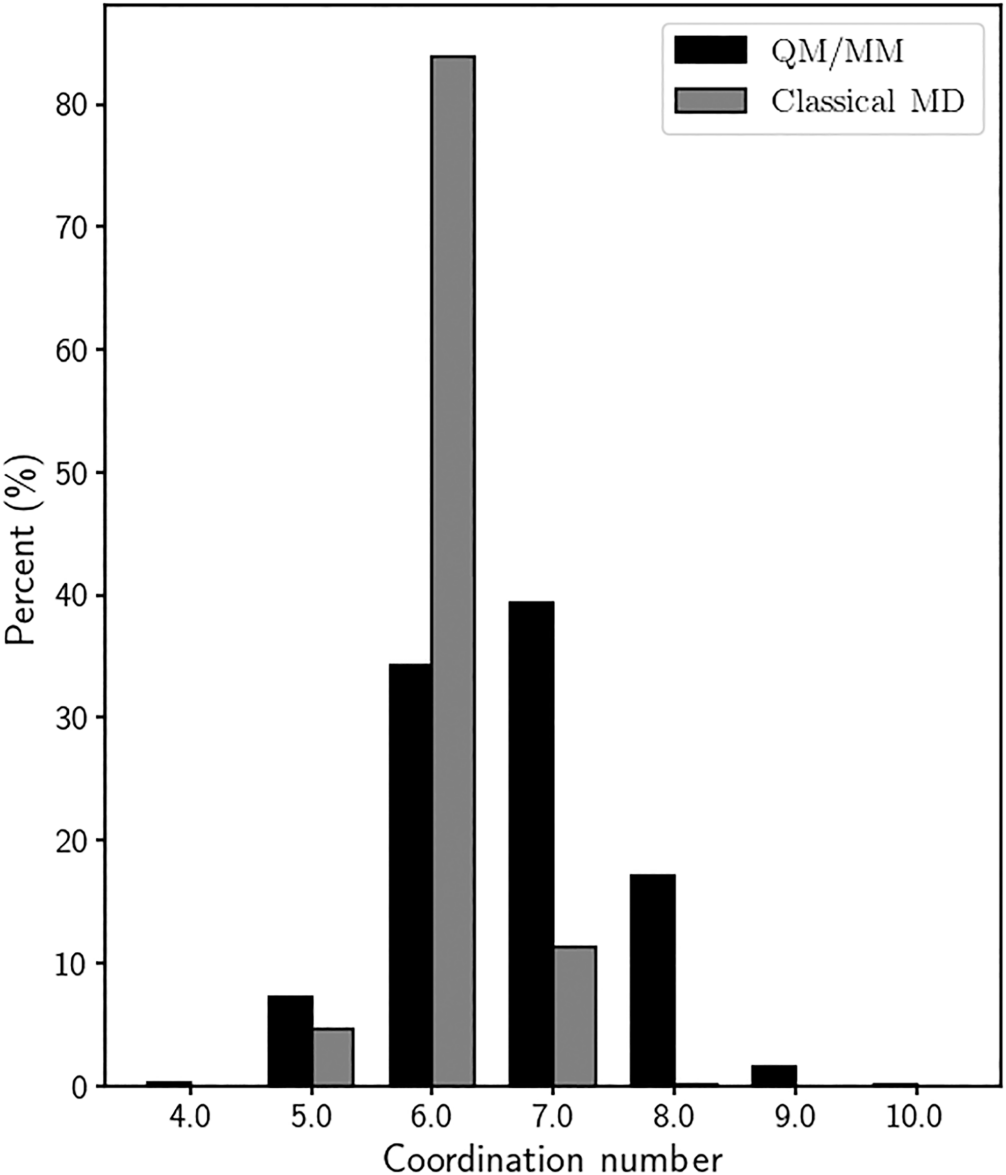
Coordination number distributions calculated up to the first minimum of the F^–^···O RDF in F^–^···H_2_O interactions.

**Figure 3:**
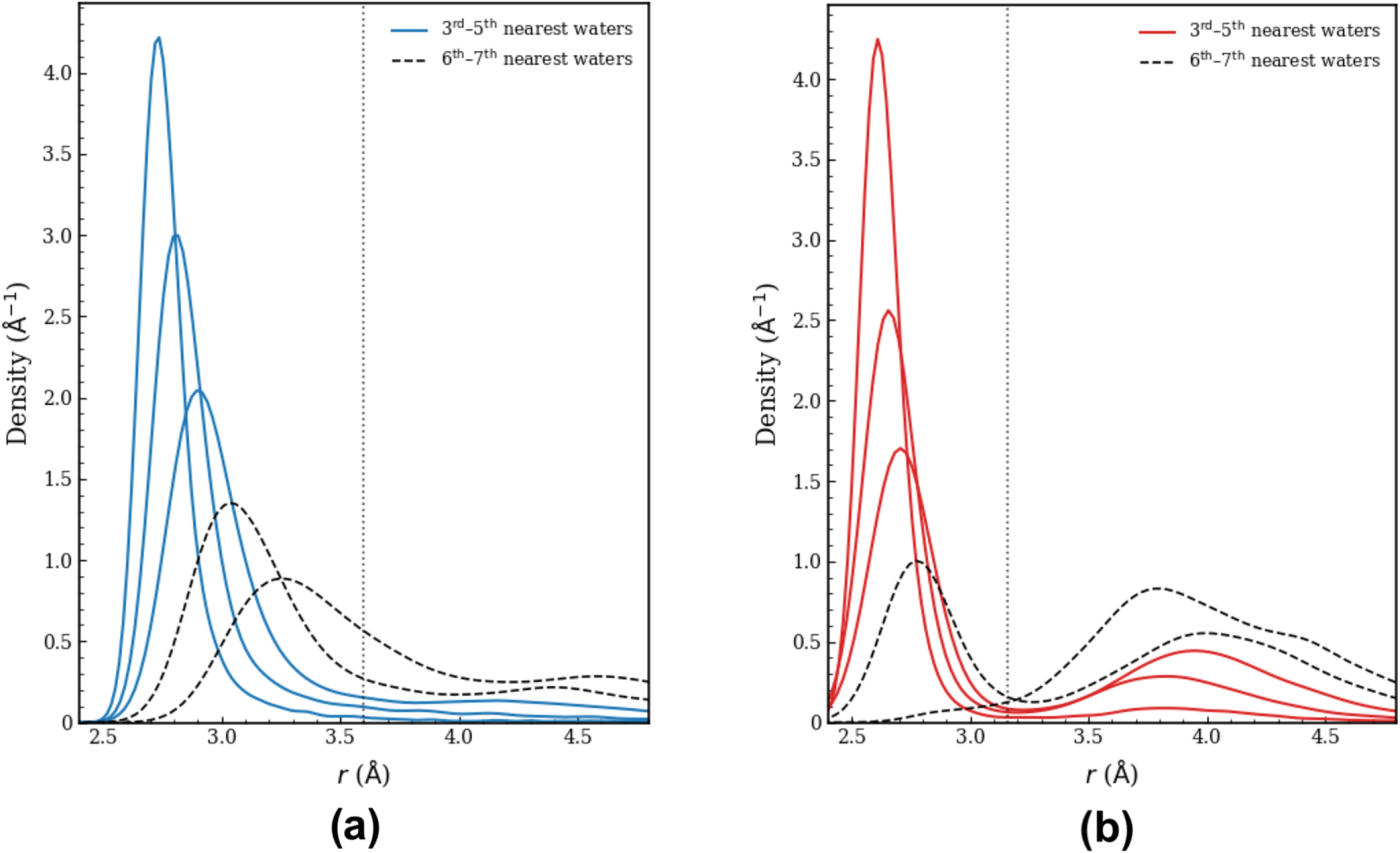
Distance distributions between the F^–^ anion and its first seven coordinating water molecules obtained from adaptive (a) hybrid QM/MM and (b) classical MD simulations. Solid lines represent the third to fifth nearest water molecules, while dashed black lines indicate the sixth and the seventh nearest molecules. The vertical dotted line denotes the first minimum of the F^–^···O radial distribution function. While classical MD shows a clear tendency for the seventh molecule to reside in the second solvation shell leading to CN = 6, QM/MM results exhibit a less dispersed distribution with CN = 7.

We employed adaptive QM/MM simulations, maintaining initially four fixed water molecules within the QM region along with one mobile water molecule in the transition region. In this active zone, a distance-based criterion determines whether a molecule is treated at the QM or MM level. To verify if the extra molecules in the first coordination sphere were an artifact of the simulation setup, we extended the QM region to include one mobile plus six fixed H_2_O molecules. These extended calculations yielded consistent structural results in the F^–^···O and F^–^···H RDFs, converging to NC = 7. As Hofer previously pointed out,^85^ the pronounced tailing in first-shell ligand distributions can make the determination of coordination numbers based strictly on radial cutoffs problematic. In this work, we analyzed the distance distributions of the seven nearest water molecules to the F^–^ center. We observed a progressive broadening and decrease in peak intensity for the successive water neighbor distributions. While the innermost molecules exhibit sharp and narrow profiles, a clear transition toward more dispersed curves is evident starting from the fifth neighbor. Notably, the majority of the probability density for the sixth and seventh neighbors remains within the first minimum of the F^–^···O RDF. These observations suggest a highly dynamic first hydration shell where a core of tightly bound water monomers is surrounded by additional H_2_O molecules that, while remaining within the coordination limit, exhibit a more labile character and are prone to exchange with H_2_O molecules in other hydration shells (see Figure 3). Figure 4 displays the O···F^–^···O angular distributions, calculated up to the first minimum of the F^–^···O RDF. The octahedral arrangement is clearly recognized in the classical potential simulation, by the two pronounced peaks between 70–110 and 150–180°. The QM/MM calculations of the ···F^–^···O angle show a first peak between 50 and 100° and a broader, less pronounced peak around 120–140°. These maxima in the angular distribution are smaller than those of the reference octahedral configuration from classical MD, reflecting an increased coordination number, as previously discussed. In addition, the hybrid calculations lead to a more disordered configuration of water molecules in the first coordination shell, as shown in Figure 4.

**Figure 4:**
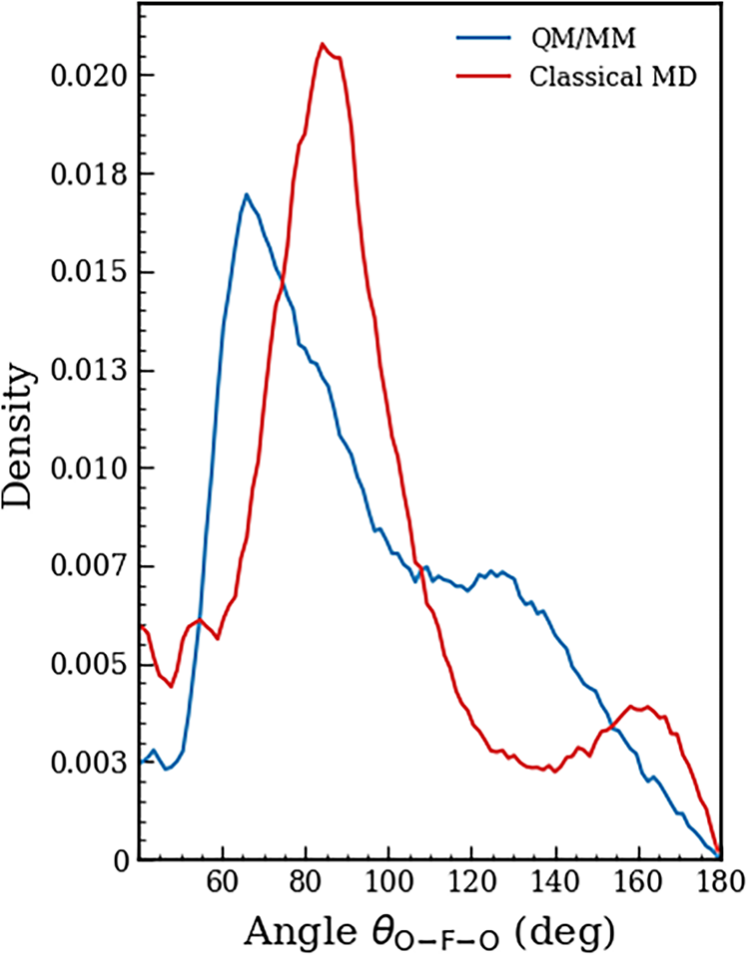
O···F^–^···O angular distribution calculated up to the first minimum of the F^–^···O RDF in the F^–^···H–OH interaction.

In summary, the distinct structural descriptions provided by classical and hydrated QM/MM simulations of the hydrated F^–^ anion underscore the necessity of a quantum mechanical treatment for this system. While classical MD (relying on fixed point charges and rigid atomic sizes) imposes a strict octahedral geometry, the QM/MM approach reveals a more flexible hydration shell capable of accommodating a seventh water molecule. This structural plasticity is intrinsically linked to the significant covalent component of the F^–^···H_2_O interaction identified by our IQA analysis, a feature that classical potentials are inherently unable to reproduce. Consequently, including explicit electronic structure effects is indispensable to accurately capture the subtle interplay between covalency and hydration dynamics in the fluoride anion.

### 4.2 ZF in NPL4

The discussion of the results concerning the ZF in NPL4 is organized in four parts, each addressing a distinct stage in the construction, validation, and assessment of the demarcation of the quantum-mechanically addressed region in the hybrid QM/MM simulations for metalloproteins examined in this paper. First, we report the characterization of the covalent and ionic contributions for the interactions involved in the coordination of Zn^2+^ within the NPL4 protein via QTAIM and IQA analyses. As mentioned before, these analyses comprise the basis of the diagnostic methodology put forward in this paper to delimit QM regions in QM/MM calculations. Second, we discuss the performance of hybrid simulations using different QM regions. By these means, we were able to characterize the QM region demarcated on the basis of covalency as the one which provides the best results when contrasted with electronic structure theory and structural experimental data. Third, we compare the results of classical force field simulations with those of hybrid QM/MM calculations, and we highlight systematic deviations in the coordination geometry and the distribution of metal–ligand distances of classical MD computations. Fourth, we assess how hybrid simulations recover correct coordination numbers even when initialized with incorrect octahedral geometries which result from classical MD, to finally summarize the methodological workflow and discuss the potential generalization of our diagnostic to other metalloproteins.

### 4.3 QCT Analyses and Delimitation of the QM Region

In order to identify a chemically meaningful quantum region for hybrid QM/MM simulations of the addressed ZF, we applied the theoretical framework of QCT to investigate the chemical bonding scenario within the zinc coordination environment in the NPL4­(Zn^2+^)_129–151_ domain. We used the QTAIM and IQA methods of wave function analysis to decompose the interaction energy between Zn^2+^, its surrounding amino acids, and solvating water molecules into classical (i.e., ionic) and exchange–correlation (i.e., covalent) contributions. For this purpose, we considered the four pockets previously defined in Section 3.3. As shown in Figure 5, both energy components are strongly attractive within Pocket 1, which comprises the metal center together with the Cys137, His139, Cys145, and Cys148 amino acid residues. The IQA exchange–correlation term, associated with covalent character, reaches values between −60 and −80 kcal mol^–1^ for the Zn–S and Zn–N interactions, respectively. The classical electrostatic interaction, while also attractive in this inner region, spans a broader range and reaches a minimum of approximately −120 kcal mol^–1^.

**Figure 5:**
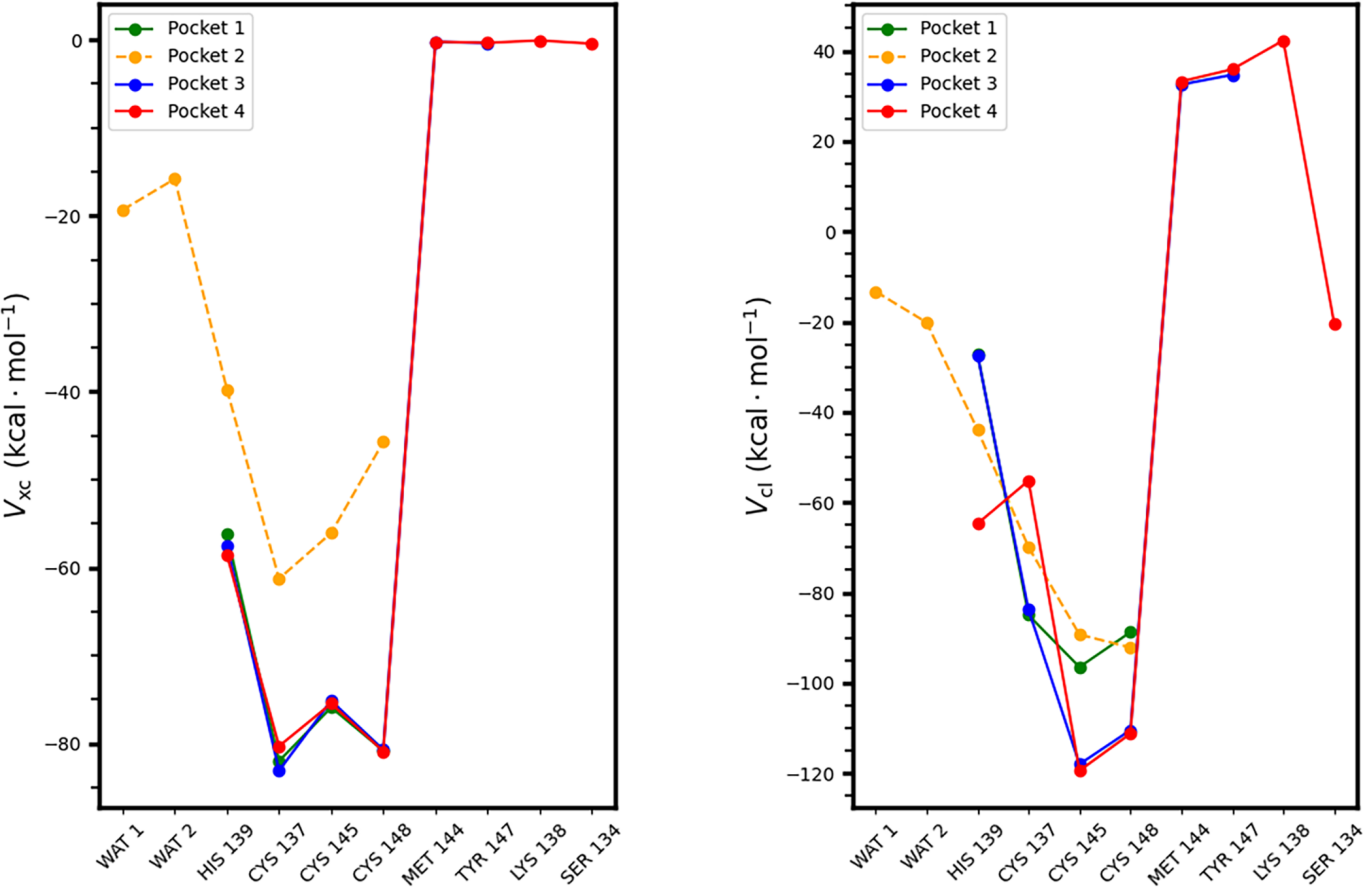
(Left) Exchange–correlation and (right) classical components of the IQA interaction energies between Zn^2+^ and the amino acid residues as well as solvating water molecules in Pockets 1–4 (defined at the end of Section 3.3) within the ZF of the NPL4 protein.

In Pocket 2, the covalent components of the Zn^2+^···OH_2_ interactions are notably weaker (−15.82 and −19.35 kcal mol^–1^), less than half the magnitude of the weakest Zn–ligand interaction observed with His139 (−39.74 kcal mol^–1^). Although the Zn^2+^···OH_2_ contact retains partial covalent character, its contribution alone is insufficient to stabilize the octahedral coordination environment. Table 1 reports that for a fully hydrated Zn^2+^ ion coordinated by six water molecules, the average covalent contribution per Zn^2+^···OH_2_ interaction is significantly stronger (up to −58 kcal mol^–1^). Furthermore, within the ring water hexamer, individual H_2_O···OH_2_ interactions can contribute up to −27.53 kcal mol^–1^. In other words, the covalent contribution in Zn^2+^···OH_2_ interactions within the zinc finger environment are substantially smaller in magnitude than those corresponding to all the other interactions shown in Table 1, indicating that water has a substantially smaller covalent contribution in its interaction with Zn^2+^ in the ZF than in aqueous solution, and it is even smaller than that of H_2_O molecules in water clusters. We want to emphasize that although the O···O distance in the water hexamer (2.68 Å) is longer than the Zn^2+^···O distance in the ZF (2.47 and 2.50 Å), the Zn^2+^···OH_2_ interaction in the ZF still shows a notably weaker covalent character. These findings suggest that the inclusion of water molecules in the QM region should be guided by quantitative interaction energies rather than solely by geometric criteria, as these guidelines might lead to different conclusions. The analysis of the data in Table 1 points to the exclusion of water molecules from the QM region in MD QM/MM simulations. Although it would be desirable that one could foresee that the exclusion of the explicit solvation water molecules is a transferable effect to other Zn-finger proteins, we believe that this circumstance is unfortunately not the case. As indicated in the first row of Table 1, it is feasible for the interaction between a Zn^2+^ ion and water molecules to exhibit a relevant covalent component. Therefore, it is our opinion that the inclusion or exclusion of water molecules of other Zn-finger proteins must be determined for each individual case.

When we considered those amino acid residues which are not in Pockets 1 and 2, namely, Lys138, Tyr147, Ser134, and Met144, we noted that the IQA exchange–correlation contribution for the interaction between the Zn^2+^ center and these amino acids decays rapidly toward zero, a result which indicates a negligible covalency among these species. We noted the same trend by considering the approximation to *V*_xc_^AB^ in eq 9 as reported in Figure S2. Likewise, the classical contribution to the IQA interaction energy between Zn^2+^ and the residues within Pocket 1 is attractive, whereas the same term becomes repulsive for Lys138, Tyr147, and Met144 in Pockets 3 and 4.

These results establish that only the first coordination shell around Zn^2+^ without solvating water molecules exhibits significant covalent character in its bonding with this metallic center, while the interactions with more distant amino acid residues are predominantly electrostatic. Based on this analysis, we selected the atoms in Pocket 1 as a suitable quantum mechanical subsystem for hybrid QM/MM simulations of the ZF within NPL4. This region comprises all residues with non-negligible IQA exchange–correlation interactions, thereby ensuring an accurate quantum mechanical description of bonding while maintaining computational efficiency by excluding second-shell amino acids and solvating water molecules that can be treated classically. This pocket is therefore adopted as the reference QM region for the analysis of ZF in NPL4 throughout this work. Before we examine the comparison between hybrid QM/MM and classical MD simulations for the ZF in NPL4, we point out that the data in Table 1 and Figure 5 suggest that reasonable criteria for the inclusion of a moiety in a QM region are (i) that its covalent contribution with the most relevant part of the QM system is similar or more significant than the electrostatic one, i.e., $\left|V_{\mathrm{xc}}^{\mathrm{GH}}\right|≳\left|V_{\mathrm{cl}}^{\mathrm{GH}}\right|$ and (ii) that this covalent component makes an important energetic contribution. The data contained in Table 1 and Figure 5 suggest $\left|V_{\mathrm{xc}}^{\mathrm{GH}}\right|≳35\;\mathrm{kcal}\;{\mathrm{mol}}^{-1}$ .

### 4.4 Performance of Hybrid Simulations Using Different QM Regions

So as to assess the suitability of this choice for the QM subsystem of our QM/MM calculations, we defined three candidate quantum regions, as illustrated in Figure 6. These regions are not to be confused with Pockets 1–4 defined in Section 3.3. Region I is a reduced model in comparison with Pocket 1, which excludes the residue His139, i.e., Region I includes the metal center along with the amino acids Cys137, Cys145, and Cys148. On the other hand, Region II corresponds to the proposed optimal QM region based on the analysis of the covalency of the coordination sphere of Zn^2+^ as described above. In other words, Region II comprises the Zn^2+^ ion as well as Cys137, His139, Cys145, and Cys148. Finally, Region III extends Region II by including additional nearby residues beyond the first coordination shell. That is to say, Region III encompasses those species in Region II along with Leu136, Met144, Glu146, and Ser149. We performed hybrid QM/MM simulations with these three regions to determine how the inclusion or exclusion of specific amino acids influences the structure of the coordination environment around Zn^2+^. Section 4.4 addresses hybrid QM/MM simulations in which the QM region includes explicit solvation water molecules for the same purpose of evaluating the suitability of the chosen QM region.

**Figure 6:**
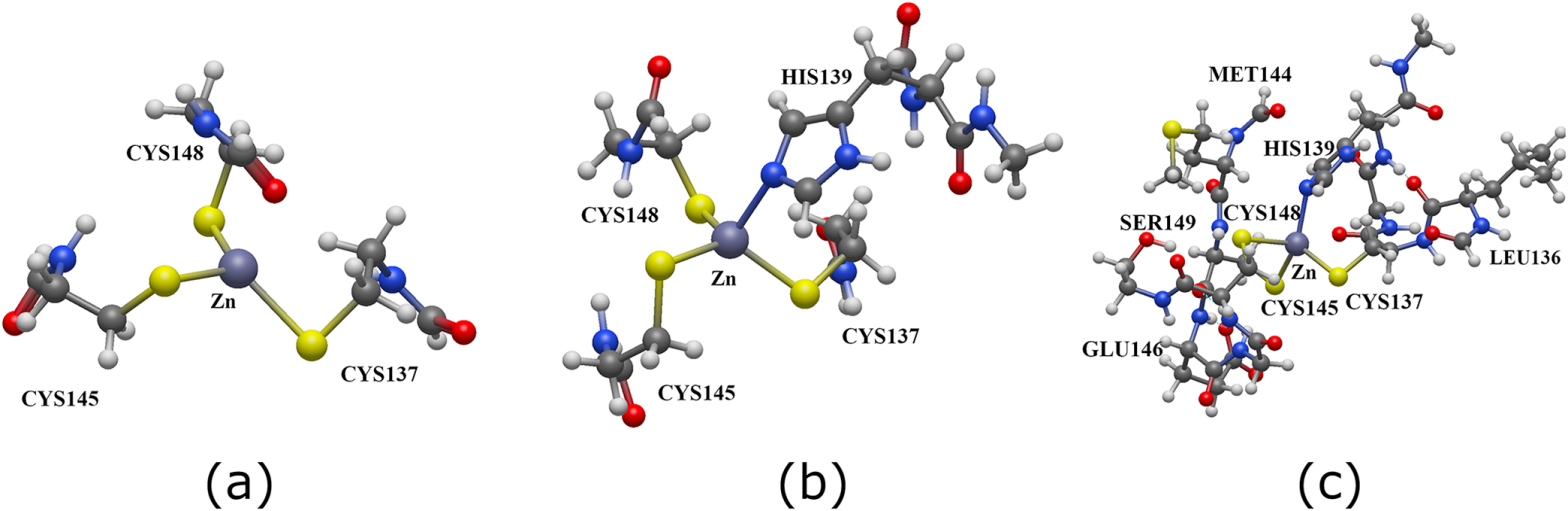
Quantum mechanical regions defined for hybrid QM/MM simulations of the NPL4 zinc finger, considered in this investigation. (a) Region I includes the Zn^2+^ metal center and the three coordinating cysteine residues, Cys137, Cys145, and Cys148, but it excludes His139. (b) Region II, as determined by examining the chemical bonding scenario with the QTAIM and IQA methods of wave function analyses, comprises those species in Region I along with His139. (c) Region III extends Region II by incorporating additional nearby residues beyond the first coordination shell, i.e., Leu136, Met144, Glu146, and Ser149. Atom color representation code: sulfur appears in yellow, nitrogen in blue, hydrogen in white, carbon in gray, and oxygen in red.

Figure 7 shows the distance distributions between Zn^2+^ and its coordinating ligands in each of the three QM regions of Figure 6. We emphasize that only simulations using Region II, which includes the Zn^2+^ metal center along with Cys137, His139, Cys145, and Cys148 identified as covalently bound to the metallic ion via the QTAIM and IQA analyses, yield distance distributions for Zn–S and Zn–N interactions that closely match those from geometries optimized with the approximation B3LYP/Def2-TZVP. The SHAPE measure quantifies the deviation of a set of atoms from an ideal reference geometry, e.g., the tetrahedron (denoted as T-4 by the IUPAC), using a single dimensionless parameter, i.e., the Continuous Shape Measure (CShM). This parameter evaluates the total distance between the atomic positions and those of the ideal shape after an optimal alignment in terms of position, size, and orientation. A value of *S* = 0.00 indicates a perfect correspondence with the reference shape. Small values of *S* (*S* < 1.0) correspond to minor deviations, while values of *S* exceeding one denote substantial departures from the reference geometry.^86^ In this work, we obtained a CShM score of *S* = 0.81 for the tetrahedron geometry, while the vacant trigonal bipyramid, seesaw, and square geometries presented significantly higher scores of *S* = 2.43, 6.70, and 27.76, respectively. These results indicate that the coordination structure around Zn^2+^ from QM/MM simulations not only reproduces the interatomic distances predicted by QM calculations, but also exhibits a tetrahedral coordination geometry consistent with crystallographic data.^60^ Moreover, the Zn–S distances for the three sulfur atoms are sharply peaked around 2.3 Å (Figure 7a–c), and the Zn–N distribution shows a well-defined maximum near 2.0 Å (Figure 7d), consistent with electronic structure theory and experimentally determined values.^60^

**Figure 7:**
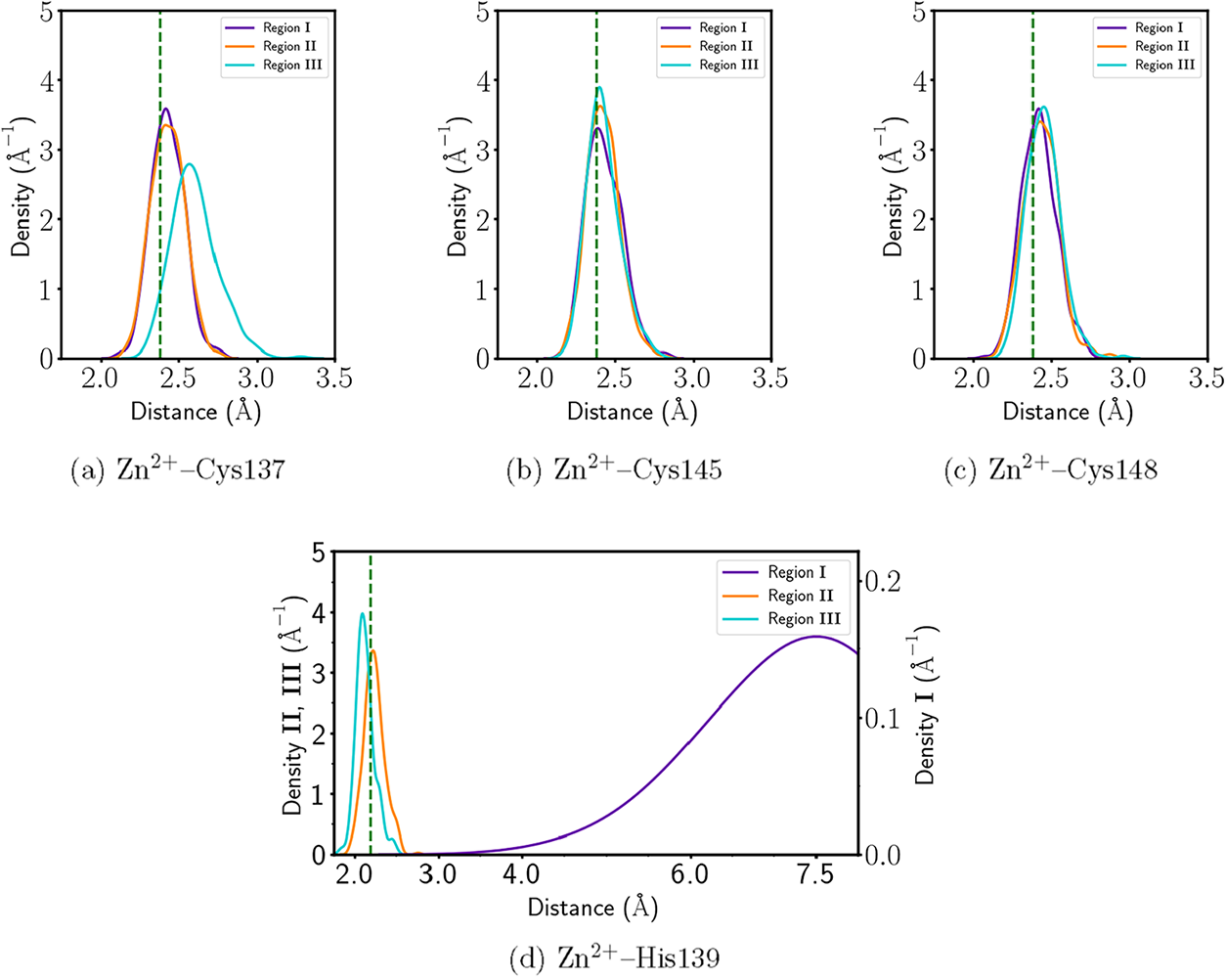
Distance distributions between the Zn^2+^ ion and its coordinating residues for the ZF within the protein NPL4 obtained from QM/MM simulations for the three QM regions defined in Figure 6, with the CHARMM36m force field and the GFN2-xTB semiempirical method as the quantum chemical approximation. Reference values from electronic structure calculations (B3LYP/Def2-TZVP) are shown with dashed green lines. Panels (a)–(c) address the Zn^2+^–S interactions for Cys137, Cys145 and Cys148 respectively. Ditto for panel (d) and the Zn^2+^–N interaction for His139. The Zn–N distance in panel (d) for Region I is around 7.5 Å, which is too long to maintain Zn–N coordination, and therefore His139 is prevented from forming a chemical bond with Zn^2+^, by considering Region I as this QM subsystem.

On the contrary, Region I, which excludes His139 from the QM region, fails to properly describe the Zn–N coordination. The Zn–N distance distribution for this region is broad and shifted toward longer distances, with a first peak near 7.5 Å as it can be seen in Figure 7d. Classical force fields do not accurately represent coordination interactions, as they lack an explicit description of electron transfer, induced polarization, and the significant covalent nature of the metal–ligand bond. Most importantly, this result reflects the inability of the MM methodology to stabilize the Zn–N interaction in the absence of a quantum description. On the other hand, the Zn–S distances remain relatively well-described, indicating that a minimal QM region that rules His139 out is sufficient to capture the sulfur coordination to Zn^2+^. Nevertheless, the same region is inadequate to maintain the correct nitrogen coordination to the same metallic center. Therefore, Region I fails to correctly reproduce the coordination sphere around the Zn^2+^ ion within NPL4.

Likewise, QM/MM simulations considering quantum subsystem Region III, which includes additional second-shell residues, do not yield an improved agreement with respect to structural parameters computed with electronic structure theory (B3LYP/Def2-TZVP). On the contrary, the Zn–S (Cys137) distance distribution for Region III (Figure 7a) becomes broader, and it shifts away from the corresponding QM value compared to the corresponding results computed with Regions I and II. This decrease in the quality of the results indicates that the extension of the QM region beyond the first coordination shell may introduce either an excess of flexibility in the system or destabilizing boundary effects likely due to imbalanced electronic interactions at the QM/MM interface.^87^ The results obtained with QM/MM computations using Region III as the quantum mechanical subsystem reveal that the definition of the QM region without a systematic and justified selection criterion can make the overall calculation error-prone. This observation does not imply that enlarging the QM region is intrinsically detrimental, but it rather shows that an inadequately defined boundary can introduce artifacts, regardless of the size of the QM region. Indeed, the expansion of the QM region without proper sampling or carefully defined selection criteria can spuriously intensify the influence of distant charged residues of the MM region on the QM subsystem.^88^ Finally, the selection of QM atoms based solely on distance criteria is often inadequate, as residues that are not typically chosen using chemical intuition might be necessary for a correct convergence of energetic and structural properties.^20^

To inquire further into this problem, we recall that we employed the H-link atom scheme implemented by default in AMBER 24,^77^ in which an additional hydrogen atom is placed to cap an eventually broken covalent bond at the QM/MM interface. The introduction of such an auxiliary atom may lead to several well-known issues: (a) each link atom introduces structural degrees of freedom that do not exist in the real system; (b) it occurs charge imbalance; (c) the link atom and therefore, the QM electron density is placed very close to frontier MM atoms (M), and thus the point charges located on M may overpolarize the QM density. To mitigate point (a), the coordinates of the link atom are expressed by default in terms of those of the parent atom so that no additional internal degrees of freedom contribute explicitly to the energy or forces of the system. Regarding point (b), i.e., the occurrence of charge imbalance, we performed hybrid QM/MM calculations employing a charge-conserving scheme in the presence of link atoms. In this approach, when the QM region is defined, the atomic charges of the QM atoms and any MM atoms involved in link bonds are set to zero and the net charge of the QM region is explicitly constrained and conserved throughout the simulation. As a result, the QM region cannot gain or lose net charge during the QM/MM calculations, preventing any artificial charge imbalance between the QM and MM subsystems. Any observed effects, therefore, arise from a redistribution of the QM electron density rather than from an incorrect total charge. Nevertheless, concerning point (c), there is a tendency of the QM density to be overpolarized by the fixed MM point charges. This artifact is always present to some extent when a point charge interacts with a polarizable electronic distribution, but it becomes more severe as the point charge approaches the QM density, especially when the QM density is polarizable.^89^ The problem is particularly critical at the boundary when link atoms are present because they are positioned in close proximity to the frontier MM atom. Because MM atoms carry fixed partial charges, the occurrence of charged amino acids close to the boundary can introduce strong electrostatic interactions that propagate into the QM region. In our system, Region III includes a Glu^–^ residue located close to the QM/MM boundary. The above-mentioned defective behavior of the Zn–Cys137 distance distribution can therefore be attributed to additional perturbations of the QM region geometry arising from the strong electrostatic interactions associated with this Glu^–^ residue at the interface.

Overall, these results show how Region II offers the best balance between computational cost and physical accuracy by maintaining correct coordination number and geometry as well as distance distributions while avoiding the artifacts associated with both under- and overinclusion of residues in the QM region. Moreover, this analysis indicates the validity of the choice of the QM region based on the assessment of covalency derived from the IQA and QTAIM methods of wave function analysis, and it establishes Region II as the most reliable reference QM region for the subsequent hybrid QM/MM simulations of the ZF in NPL4 addressed below.

### 4.5 Comparison of Classical Molecular Dynamics and Hybrid Simulations in the ZF of NPL4

In order to assess the performance of classical molecular mechanics in the description of the Zn^2+^ coordination within NPL4, we compared the results of classical MD simulations performed with three standard force fields, CHARMM36m, ff99SB-ILDN, and ff19SB-GAFF—against those of hybrid QM/MM simulations using Region II as the QM subsystem. We conducted all simulations on the same NPL4­(Zn^2+^)_129–151_ domain, and we focused on Zn–S and Zn–N distance distributions and geometry coordination around the Zn^2+^ ion.

Among the tested force fields, CHARMM36m shows the largest deviation from both electronic structure calculations (B3LYP/Def2-TZVP) and experimental reference data.^60^ In particular, as shown in Figure 8a, CHARMM36m simulations exhibit a persistent tendency to incorporate two water molecules into the coordination sphere of Zn^2+^, thereby promoting a factitious octahedral geometry around Zn^2+^ instead of the expected tetrahedral configuration. This effect emerges consistently after the first nanoseconds of simulation, and it is accompanied by a systematic elongation of the Zn–S bond distances. The change in coordination geometry from tetrahedral to octahedral agrees with previous findings by Scrima et al.,^1^ who exploited the force fields CHARMM36m, ff99SB-ILDN, and ff99SB*-ILDN to study the ZF within NPL4. Likewise, ref 1 reported an artificially increased coordination number along with overestimation of the Zn–S and Zn–N bond lengths, both in the zinc domain and in the full protein using the CHARMM36m force field. These effects are associated, especially in CHARMM36m (a nonbonded model), with an overstabilization of Zn–O interactions, which favors the addition of oxygenated extra ligands (e.g., water) and alters the coordination around the Zn^2+^ metal center. This overstabilization of Zn–O interactions by the CHARMM36m force field reflects the development of its parameters for this type of interactions, which depended to a large extent upon the consideration of the [Zn­(H_2_O)_6_]^2+^ complex.^90,91^ Table 1 reports that the Zn^2+^···OH_2_ interactions in the complex [Zn­(H_2_O)_6_]^2+^ are substantially stronger than those in the ZF of NPL4 as discussed above. In contrast, the use of the ff99SB-ILDN force field in this work resulted in a stable coordination sphere around Zn^2+^. This observation is consistent with previous classical MD simulations of the ZF within NPL4 using ff99SB*-ILDN, which showed a small deviation of coordination bond lengths with respect to the reference QM distances (less than 0.2 Å).^1^ In this study, ff99SB-ILDN and ff19SB show larger deviations with respect to QM distances (about 0.5 and 0.2 Å, respectively). The tendency of AMBER force fields to produce narrow distance distributions, as shown in the bottom part of Figure S3, suggests a deep, rigid potential well that limits thermal fluctuations. Such a result might indicate an overfitted model that forces the system toward fixed, shorter equilibrium distances, potentially at the cost of structural realism. The computed values for the Zn–S distance are consistently shorter than those obtained from the crystallographic data (2.3 Å). This observation indicates that the force field favors overall structural stability over local chemical accuracy, possibly leading to systematic errors in the geometry of the coordination sphere of Zn^2+^. In short, simulations with the ff99SB-ILDN force field, shown in panels (c) and (d) of Figure 8, provide geometries closer to hybrid QM/MM computations performed with Region II, avoiding spurious hydration as opposed to the simulations performed with CHARMM36m. However, the ff99SB-ILDN force field tends to underestimate Zn–ligand bond lengths. While less severe than the shortcomings found with CHARMM36m, the deviations computed with ff99SB-ILDN and ff19SB-GAFF indicate that force fields struggle to simultaneously preserve the correct coordination geometry and chemical bond metrics in the examined systems.

**Figure 8:**
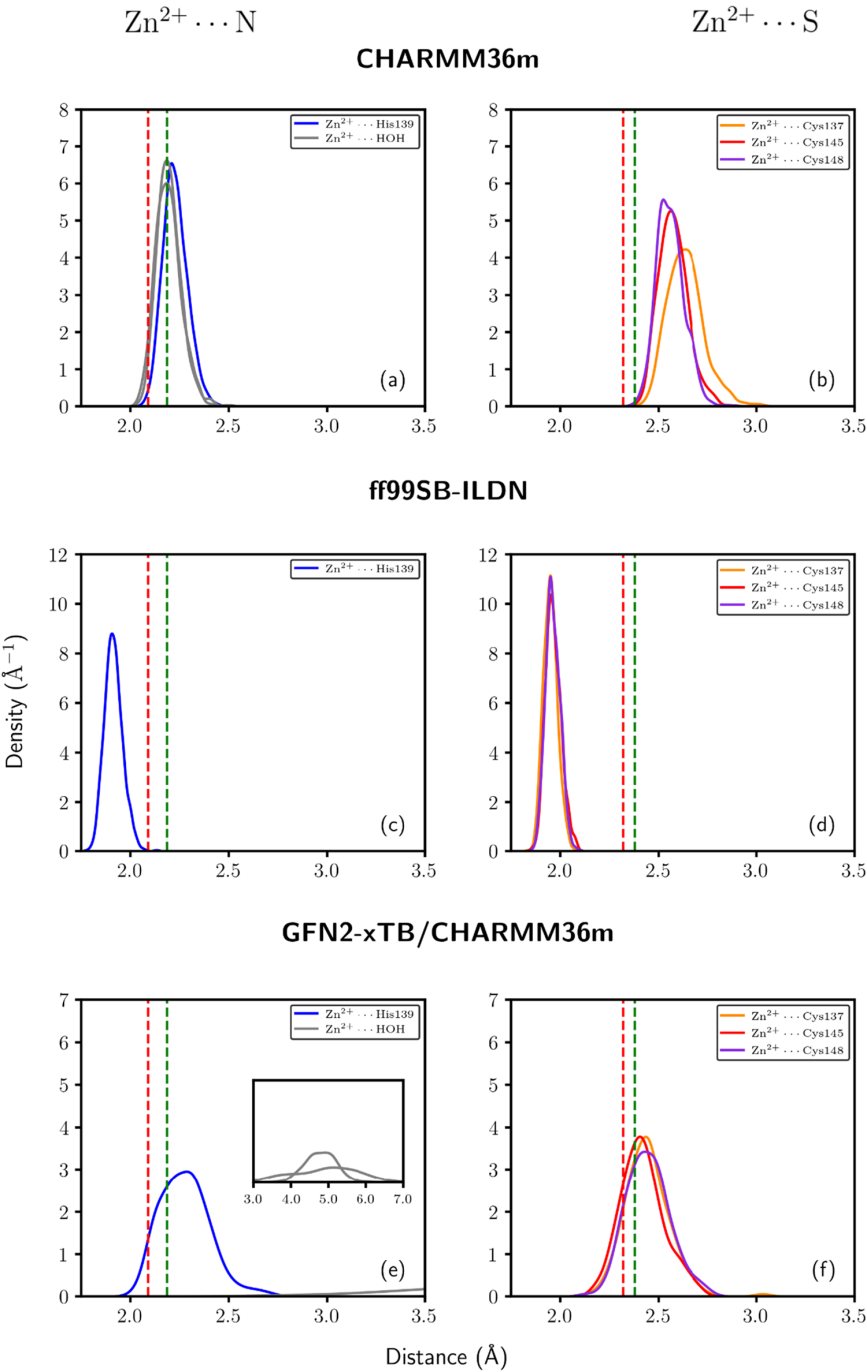
Distance distributions for Zn^2+^–ligand interactions in the ZF within NPL4 obtained from classical MD simulations using the force fields CHARMM36m (a, b) and ff99SB-ILDN (c, d). Reference hybrid GFN2-xTB/CHARMM36m simulations initialized with spurious hydrated octahedral structures obtained with classical molecular dynamics calculations are shown in panels (e) and (f). The panels on the left display the Zn^2+^–N interactions for His139, whereas those on the right address the Zn^2+^–S contacts for Cys137, Cys145, and Cys148. Green dashed vertical lines indicate reference distances from the electronic structure calculations (B3LYP/Def2-TZVP). Ditto for red dashed vertical lines and crystallographic data. CHARMM36m simulations show a tendency to incorporate water molecules into the coordination sphere of Zn^2+^, as shown in panel (a).

We consider now the chemical bonding scenario of Zn^2+^ complexes along with the force fields exploited in this investigation. The ion Zn^2+^ is a d^10^ cation with a fully filled d-shell, which indicates a spherically symmetric electron distribution and a tendency toward near symmetric coordination. Nonetheless, differences in the electronegativity and donor properties of the ligand atoms, i.e., nitrogen from histidine and sulfur from cysteine, induce strong polarization effects in the electron density around the zinc center. Such effects are reflected in the IQA analysis, where the magnitude of the covalent component of the Zn–His interaction is found to be −60 kcal mol^–1^, while that of Zn–Cys interactions is about −80 kcal mol^–1^. This asymmetry in covalent contributions strengthens the differences of Zn–ligand distances. This effect is partially recovered by classical MD. Although standard classical force fields typically use pairwise, spherically symmetric interaction potentials, which favor uniform coordination geometries, CHARMM36m is able to reproduce the asymmetry in Zn–ligand distances. However, this force field also introduces additional water molecules in the coordination sphere of Zn^2+^ as previously pointed out. In contrast, ff99-ILDN shows a tendency to treat Zn–N and Zn–S interactions similarly, resulting in a more symmetric coordination environment around the Zn^2+^ ion. Additionally, QTAIM analyses indicate that the Zn^2+^ ion carries a partial charge of approximately 1 au; hence, a substantial amount of electron density is donated from the surrounding ligands to the zinc center.

Altogether, these observations highlight two phenomena that are difficult to capture using standard classical force fields: the anisotropic redistribution of electronic density induced by ligands with different donor properties and the resulting asymmetry in coordination geometry. As already mentioned, AMBER force fields reproduce the correct coordination number of the zinc domain but show narrow distance distributions. This effect leads to restricted dynamics in the corresponding simulations, and it likely occurs because these force fields are parametrized to reproduce fixed reference values. In contrast, QM/MM calculations display broader distance distributions, with peaks close to reference values. These broader displacements are important, especially in chemical reactions, where atomic movements are essential for the formation and breaking of chemical bonds. While the force field CHARMM36m can approximate the asymmetric geometry around the Zn^2+^ ion due to its parametrization strategy, only QM/MM hybrid calculations are able to accurately describe both the electronic structure and geometry of the first coordination sphere around the Zn^2+^ center.

### 4.6 Hybrid QM/MM Simulations Initialized with MD Structures

In order to evaluate the robustness of the QM region for the ZF in NPL4 put forward herein, we examined whether it could recover the correct coordination number (from 6 to 4) around the Zn^2+^ center when simulations were initialized from distorted structures computed with classical MD simulations. Specifically, we used final snapshots from classical MD CHARMM36m calculations in which two water molecules had entered the metal coordination sphere, leading to an artificial octahedral arrangement around Zn^2+^, as starting points for hybrid QM/MM computations, including Region II and two additional water molecules as the QM subsystem. We report the corresponding results in panels (e) and (f) of Figure 8. In all cases, the hybrid simulations reverted to a four coordination number within the first few nanoseconds. The Zn–O distance distributions broadened significantly, with coordination waters moving away from the metal center, while the Zn–S and Zn–N distances returned to values consistent with DFT electronic structure calculations and experimental data. This behavior confirms that the hybrid scheme is capable of correcting some artifacts induced by force fields, selectively stabilizing chemically meaningful interactions while disfavoring spurious coordination events. However, the coordination geometry changes from an octahedron to a vacant trigonal bipyramid with a CShM score of 0.95, whereas the tetrahedral geometry shows a corresponding higher score of 4.48.

In order to inquire further into this issue, we carried out geometry optimizations of Region III plus two water solvation molecules with the GFN2-xTB approximation. We observed that when the water solvation molecules are close to the metal center, then the minimal energy structure may certainly have an octahedral geometry. Therefore, the approach of solvation of water molecules can indeed distort the geometry around the metallic ion. However, as revealed by the QM/MM hybrid simulations, the water molecules are expelled throughout the evolution of the system. In short, the distortion from the tetrahedral geometry mentioned above is physically plausible when solvation H_2_O molecules appear in the vicinity of the Zn^2+^ center, but these species are eventually forced out of the coordination sphere of the metal center.

Additionally, hybrid QM/MM simulations starting from an artificially constructed octahedral configuration exhibit instabilities due to water molecules approaching the QM/MM boundary. Throughout the simulations, the H_2_O monomers drifted into the QM/MM boundary region, resulting in an exchange between the QM and MM water molecules. This change in the physical description of individual atoms as they cross the boundary can introduce significant discontinuities in the potential energy surface, severely affecting the convergence of the quantum mechanical calculations involved in hybrid QM/MM simulations.^92^ In order to mitigate this effect, we focused our analysis on short periods of time during which water molecules remained sufficiently distant from the QM/MM boundary. Within these carefully selected intervals, the hybrid simulations remained stable and consistently corrected the coordination number around the Zn^2+^ center.

These findings illustrate the fact that the hybrid QM/MM model is capable not only of preserving accurate metal–ligand interactions but also of dynamically correcting structural distortions, e.g., the coordination number introduced by classical MD calculations. However, the final coordination geometry remains sensitive to the initial configuration for such hybrid QM/MM calculations. While classical force fields show limited transferability in systems with strong metal–ligand covalency, often leading to incorrect coordination geometries or spurious ligand binding, the hybrid QM/MM simulations using Region II as the QM subsystem consistently correct, at least to some extent, the structure and bonding patterns of Zn^2+^. These observations indicate the utility of the delimitation of a covalency-informed QM region and underscore the importance of incorporating data from electronic structure theory and wave function analysis in the modeling of metalloproteins with complex coordination chemistry.

## 5 Conclusions

This study introduces a systematic protocol for the construction and validation of QM regions in hybrid QM/MM simulations regarding the aqueous solvation of ions and metalloproteins. Our approach relies on quantum chemical topology analyses, more specifically on the QTAIM and IQA theoretical frameworks, to quantify the covalency of different chemical interactions. By applying this protocol to the hydration of the F^–^ anion, we showed that the F^–^···H_2_O interactions have an important covalent contribution. Hence, the inclusion of the first solvation shell of F^–^ in the QM region of hybrid QM/MM simulations results in important differences concerning the coordination number and radial distribution functions. Likewise, the same protocol exploited to the ZF domain of the NPL4 protein allowed us to demonstrate that the QM region selected based on contributions of covalency achieves an optimal balance between computational efficiency and chemical accuracy. The hybrid QM/MM simulations with the QM region as determined via the examination of the chemical bonding scenario of the metal ion Zn^2+^ preserve distance distributions, accurately reproducing geometries and structural parameters from experimental and electronic structure calculations. This region excludes amino acid residues and solvating water molecules with negligible covalency, confirming that a minimal QM description in QM/MM calculations is adequate when guided by a thorough examination of the chemical bonding scenario of the system of interest. Furthermore, hybrid QM/MM simulations having a QM region determined by our QCT analyses, corrected some structural distortions such as coordination number introduced by classical force fields, highlighting the limitations of conventional MD in systems with strong metal–ligand covalency. The diagnostic proposed in the present article follows a workflow (analysis of covalency, delimitation of the QM region, and validation via hybrid QM/MM simulations) that offers a transferable strategy to model metalloproteins different from NPL4. This methodology is especially well suited to cases involving borderline covalent coordination or ligand environments that are difficult to characterize. The demarcation of the QM region based on the analysis of the chemical bonding scenario as presented herein, as opposed to the determination of the same area via arbitrary distance cutoffs or residue identities, contributes to the reliability and interpretability of hybrid QM/MM simulations. Future applications may support the design of more accurate QM/MM simulations in inorganic chemistry and of models of metalloproteins in bioinorganic and medicinal chemistry.

## Supplementary Material



## Data Availability

The data supporting this work are contained in the ZENODO repository (10.5281/zenodo.15778384).

## Footnotes

a The comparison between QTAIM/IQA and CSA/FSA regarding computational efficiency indicates that CSA/FSA offers a significantly lower computational cost compared to the IQA energy decomposition exploited in this work. The better efficiency of the CSA and FSA approaches stems from the fact that these methods rely on single-point QM/MM calculations to obtain the necessary data (atomic charges and Fukui functions) to perform these analyses. Although these single-point calculations might involve hundreds or even a thousand of atoms in the QM region, these computations are much faster than those required in the QTAIM and IQA methods, which require the integration of scalar fields derived from the electronic wave function over typically very irregular regions, i.e., the QTAIM atoms.^34^

b This statement holds for all simulations except for the hybrid QM/MM hybrid calculations that started from the last frame of a CHARMM36m classical trajectory to investigate the influence of solvating water molecules on the coordination geometry of the Zn^2+^ cation, as discussed in Section 4.6.

## Notes

PDB and xyz files of the QM regions considered in this investigation; approximation to the exchange–correlation energy in Pocket 3 using eq 9; and distance distributions for Zn^2+^ ligand using hybrid simulations (PDF)
