# Supplementary material for: Quantum Chemical Topology Analysis of Covalent Interactions in the Hydration of F– along with the Zinc Finger of NPL4 and Its Application to the Delimitation of QM/MM Boundaries
Source: ACS Omega. 2026 Mar 10;11(12):18970–86. doi: 10.1021/acsomega.5c11425 (PMC13044629; doi:10.1021/acsomega.5c11425)
Supplement: Supplementary file 1 [file ao5c11425_si_001.pdf]

Quantum Chemical Topology Analysis of Covalent Interactions in the Hydration of F^- along with the Zinc Finger of NPL4 and Its Application to the Delimitation of QM/MM Boundaries

Cristian E. Bahena-Méndez[a], Humberto Saint-Martin[b], Clare M[c]Cabe[c,d], José Manuel Guevara-Vela[c,*], Tomás Rocha-Rinza[a,**]

[a]*Instituto de Química, Universidad Nacional Autónoma de México, Circuito Exterior, Ciudad Universitaria, Delegación Coyoacán C.P. 04510, Ciudad de México, México.*
[b]*Instituto de Ciencias Físicas, Universidad Nacional Autónoma de México, Cuernavaca, Morelos 62210, México*
[c]*School of Engineering and Physical Sciences, Heriot-Watt University, Edinburgh EH14 4AS, Scotland, U.K.*
[d]*Department of Chemical and Biomolecular Engineering, Vanderbilt University, Nashville, Tennessee 37235-1826, United States*

In this link

`https://doi.org/10.5281/zenodo.15778384`

the interested reader can download the PDB and XYZ files corresponding to the QM regions used in this investigation for the hydration of F^- and the zinc finger in NPL4. Ditto for selected $F^- \cdots (H_2O)_n$ molecular clusters and local minima of the molecular system comprising the metal centre Zn^{2+} and its directly coordinated residues: Cys137, Cys145, Cys148, and His139. Each amino acid was truncated at the C_α position and capped with a methyl group.

[*]To whom correspondence should be addressed: j.guevara-vela@hw.ac.uk
[**]To whom correspondence should be addressed: trocha@iquimica.unam.mx

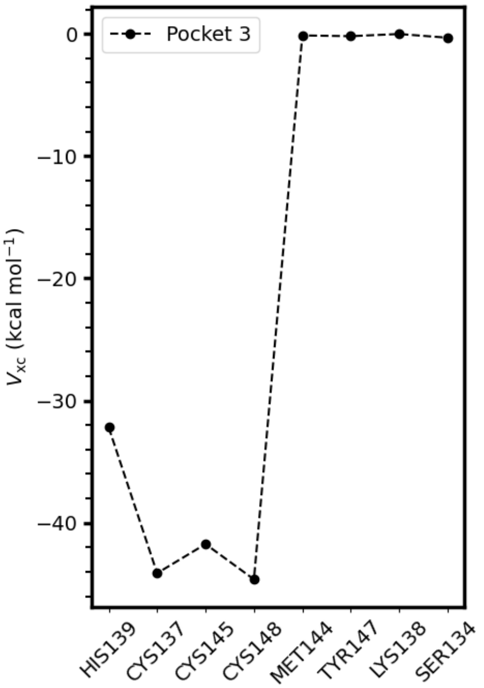

Figure S1: Approximation to the exchange-correlation component, V_{xc}, of the IQA interaction energies between Zn^{2+} and the amino acids residues constituting Pocket 3 within the zinc finger of the NPL4 protein. This approximation was computed with the aid of Equation (9) in the main body of the manuscript.

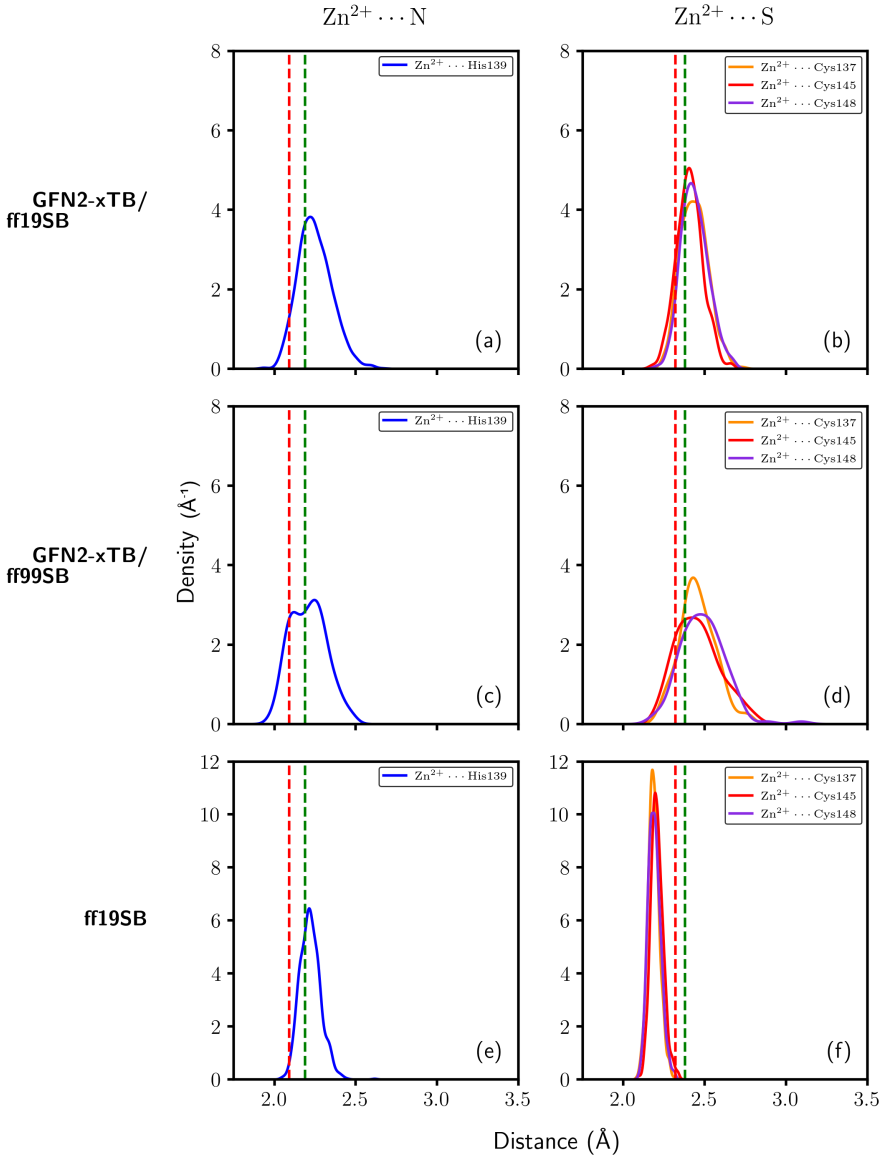

Figure S2: Distance distributions for Zn^{2+}—ligand interactions within the zinc finger of NPL4, obtained from GFN2-xTB/ff19SB ((a)-(b)) and GFN2-xTB/ff99SB ((c)-(d)) hybrid simulations. We report the results corresponding to Classical MD simulations using the force field ff19SB in panels (e)-(f). The panels of the left show the Zn^{2+}–N interactions for His139 whereas those in the right address the Zn^{2+}–S contacts for Cys137, Cys145 and Cys148. Green dashed vertical lines indicate reference distances from electronic structure calculations (B3LYP/Def2-TZVP). Ditto for red dashed vertical lines and crystallographic data.
